# Large-scale network analysis reveals the sequence space architecture of antibody repertoires

**DOI:** 10.1038/s41467-019-09278-8

**Published:** 2019-03-21

**Authors:** Enkelejda Miho, Rok Roškar, Victor Greiff, Sai T. Reddy

**Affiliations:** 1 Department of Biosystems Science and Engineering, ETH Zurich, 4058 Basel, Switzerland; 2 Institute of Medical Engineering and Medical Informatics, School of Life Sciences, FHNW University of Applied Sciences and Arts Northwestern Switzerland, 4132 Muttenz, Switzerland; 3 aiNET GmbH, c/o Switzerland Innovation Park Basel Area AG, Hochbergstrasse 60C, 4057 Basel, Switzerland; 4 Research Informatics, Scientific IT Services, ETH Zürich, 8001 Zürich, Switzerland; 5 Department of Immunology, University of Oslo, 0372 Oslo, Norway

## Abstract

The architecture of mouse and human antibody repertoires is defined by the sequence similarity networks of the clones that compose them. The major principles that define the architecture of antibody repertoires have remained largely unknown. Here, we establish a high-performance computing platform to construct large-scale networks from comprehensive human and murine antibody repertoire sequencing datasets (>100,000 unique sequences). Leveraging a network-based statistical framework, we identify three fundamental principles of antibody repertoire architecture: reproducibility, robustness and redundancy. Antibody repertoire networks are highly reproducible across individuals despite high antibody sequence dissimilarity. The architecture of antibody repertoires is robust to the removal of up to 50–90% of randomly selected clones, but fragile to the removal of public clones shared among individuals. Finally, repertoire architecture is intrinsically redundant. Our analysis provides guidelines for the large-scale network analysis of immune repertoires and may be used in the future to define disease-associated and synthetic repertoires.

## Abstract (web summary)

Analysis of large-scale immune repertoire architecture remains a challenge. Here, the authors introduce an approach to construct similarity networks from high-throughput antibody repertoire sequencing data, and show that the networks are redundant, robust and reproducible across individuals.

## Introduction

The high diversity of antibody repertoires, which is defined by the collection of an individual’s B-cell receptor (BCR) and antibody sequences, plays a major role in providing broad and protective humoral immunity. The source of antibody diversity has long been identified to be the somatic recombination V−, (D− in the heavy chains) and J-genes^1^. Additions and deletions of nucleotides at the junctions of the gene segments further increase diversity^2,3^. Antibody identity (clonality) and antigen specificity are primarily encoded in the highly diverse junctional site of recombination in the variable heavy chain, called the complementarity determining region 3 (CDR3)^4^. Thus, the similarity landscape of CDR3 amino acid (a.a.) sequences constitutes the clonal architecture of an antibody repertoire; this architecture reflects the breadth of antigen-binding and therefore correlates with humoral immune protection and function. Understanding sequence-related properties of antibodies is thus valuable for the development of novel therapeutics and vaccines^5,6^. However, due to limitations in technological sequencing depth and algorithmic advances, the fundamental construction principles of antibody repertoire architecture have remained largely unknown, thereby hindering a more profound systems understanding of humoral immunity.

**Fig. 1:**
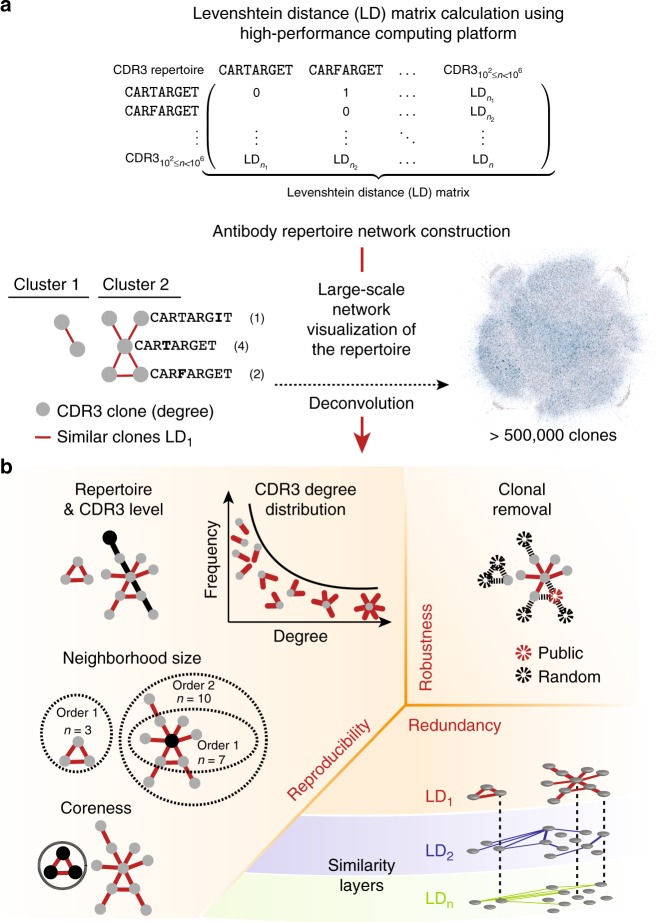
Large-scale network analysis reveals the architecture of antibody repertoires and its three fundamental principles. **a** Large-scale networks (>500,000 nodes) of antibody repertoires were constructed from the Levenshtein distance (LD, edit string distance) matrix of CDR3 clonal sequences (a.a) using a custom high-performance computing platform (see Methods). Networks represent antibody repertoires of similar CDR3 nodes connected by edges when amino acid CDR3 sequences differ by a predetermined LD. All clones of a repertoire connected at a given LD form a similarity layer (LD_n_). **b** Deconvolution of the complexity of antibody repertoire architecture was performed by quantifying (i) its reproducibility through global and clonal (local) properties or features, (ii) robustness to clonal removal and (iii) redundancy across its similarity layers in the sequence space (Supplementary Fig. 1)

Recently, selected aspects of network analysis have been employed to investigate antibody repertoire architecture in health and disease. Network analysis captures antibody repertoire architecture by representing the similarity landscape of antibody sequences as nodes (antibody clonal sequence) that are connected if sufficiently similar^7–12^ (Fig. 1a). Sequence-based networks have first been used to show immune responses defined by similarity between clones, a proxy for clonal expansion^8^. Network connectivity was later also used to discriminate between diverse repertoires of healthy individuals and clonally expanded repertoires from individuals with diseases such as chronic lymphocytic leukemia^7^ and HIV-1 infection^10^. Thus far, network analysis has mostly been utilized for visualization of network clusters^7–12^. Network visualization limits the informative graphical display of a network to a few hundred antibody clones (100% a.a. identity sequences) thereby preventing the quantitative description of immune repertoire architecture. Indeed, it has been shown that the natural antibody repertoire exceeds the informative visualization threshold (hundreds of clonal nodes) by at least three orders of magnitude^13^, a limit that previous research did not explore given the lower biological coverage. Currently, computational methods for constructing large-scale networks with more than 10^3^ nodes are not typically accessible in systems biology^14^. Furthermore, as of yet, only networks expressing clonal similarity relations of one nucleotide (nt) or one amino acid (a.a.) between sequences have been investigated^7–12^, which, considering recently discovered biases in VDJ recombination and SHM targeting^15–21^, may not be sufficient for a comprehensive immunological appreciation of repertoire architecture.

To reveal the antibody repertoire architecture by quantitative statistical analysis, we implement a high-performance computing platform for network analysis and coupled it with large-scale antibody repertoire sequencing data from murine and human B-cell subsets. This leads us to address the following key questions: (i) Is the antibody repertoire architecture reproducible across individuals? (ii) How robust is the antibody repertoire architecture to the removal (deletion) of clones? (iii) To what extent is the repertoire architecture intrinsically redundant? (Fig. 1).

## Results

### A platform for large-scale networks of antibody repertoires

The landscape of antibody clonal similarities is vast and complex; for example, on the a.a. level, the size of the distance matrix of all-against-all sequences is ≈10^12^ for a repertoire of ≈10^6^ clones (representative of murine B-cell subsets, see below). In order to extract the construction principles of antibody repertoires from such a high-dimensional similarity space, we developed a large-scale network analysis approach, which was based on representing CDR3 a.a. clones (a clone here is defined by 100% CDR3 a.a. identity) as sequence-nodes connected by similarity-edges. Specifically, we developed a computational platform that leverages the power of distributed cluster computing and computes the extremely large distance matrices required for investigating the similarity architecture of entire repertoires (≥10^6^ CDR3 a.a. sequences, Supplementary Figs. 1, 3). We performed network analysis on the a.a. level in order to emphasize information that relates to antigen-driven B-cell clonal expansion. Networks were built as follows: first the pairwise a.a. sequence similarity of all clones (distance matrix) was calculated using the Levenshtein distance (LD, Fig. 1a). Then, we built Boolean undirected networks (so called similarity layers), which are constructed such that nodes (antibody CDR3 sequences) are connected if and only if they have an LD of *n* where *n* can run from 1 through 12. For example, similarity layer LD_1_ designates the network in which CDR3 clones (nodes) are connected (via edges) if and only if, they have an LD of 1 (Fig. 1, Supplementary Fig. 1). LD measures the number of edits between sequences of arbitrary length. Therefore, CDR3 sequences did not have to be stratified by length, thus simplifying the analysis.

The network analysis of circa >10^6^ or more sequences is an intractable problem without parallel distributed computing. Our implementation utilizes the Apache Spark^22^ distributed computing framework to partition computations across a cluster of machines (Supplementary Fig. 1b). The construction of large-scale networks is computationally demanding: a large network of 1.6 million nodes (simulated strings) required 15 min if the calculation was performed simultaneously on 625 computational cores (Supplementary Fig. 1c), while the same computation would take months without parallelization. Computational costs could have been lowered substantially by performing network analysis on only a subsample of the repertoire (e.g., 10^3^ clones), as reported in previous studies^7–11^. However, extensive analysis has revealed that sub-networks are not a priori statistically representative of entire networks. For example, sub-network measurements are not always representative of key parameters such as degree distribution, betweenness, assortativity and clustering^23,24^. Thus, it was imperative to construct and analyze large-scale networks based on a similarity distance matrix that covers the clonal diversity of entire antibody repertoires.

Comprehensive biological sampling of antibody repertoires was ensured by the usage of previously generated large-scale antibody repertoire data (billions of antibody sequence reads) from human^25^ and mouse^18^ naïve and antigen-experienced B-cell populations. Data was analyzed from naïve and memory B-cells of three healthy human donors^25^, and pre-B cells (pBC), naïve B cells (nBC) and memory plasma cells (PC) isolated from 19 mice, which were stratified into one unimmunized and three immunized cohorts. The experimental design and data allowed for the assessment of antibody sequence architecture across several important parameters: (i) across species, (ii) across key stages of B-cell development, (iii) before (pBC, nBC) and after antigen-driven clonal selection and expansion (PC, memory B-cells), (iv) differences in the complexity of the protein antigen [hepatitis B surface antigen (HBsAg), ovalbumin (OVA) and nitrophenylacetyl-conjugated hen egg lysozyme (NP-HEL)], and (v) across a scale of different repertoire sizes (10^2^–10^6^ of unique CDR3 clones). The human^25^ and murine^18^ experimental data^26^ provided maximal technological and high biological coverage, enabling comprehensive assessment of the global similarity landscape and architecture of antibody repertoires^18^.

### Global patterns of antibody networks are reproducible

**Fig. 2:**
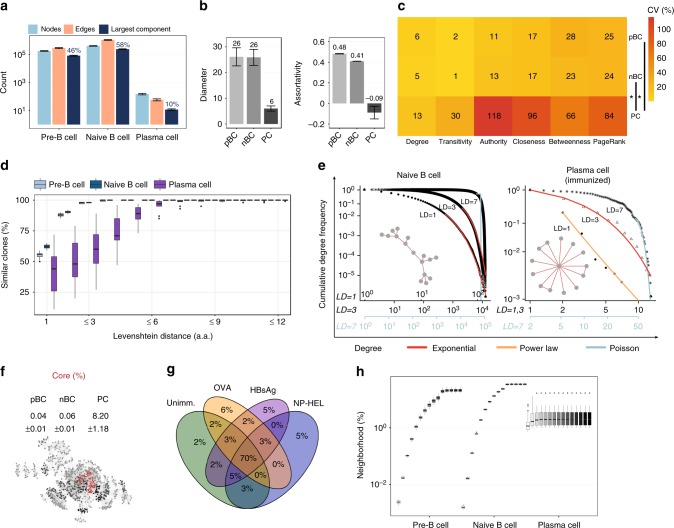
Global and clonal properties of antibody repertoire networks are reproducible. **a** Network size of antibody repertoires. The *y*-axis indicates the absolute number count of CDR3 nodes, CDR3 edges (similarities) and CDR3 clones in the largest component. The mean percentage of the CDR3s belonging to the largest component by B-cell development stage is shown on top of the dark blue bar. **b** Global properties, diameter and assortativity coefficient are shown for pre-B cells (pBC), naïve B cells (nBC) and plasma cells (PC). **c** The mean value of the coefficient of variation for clonal properties in pBC, nBC and PC repertoires. Wilcoxon test, p_pBC,nBC/PC_ < 0.05 (see Methods). **d** Percentage of clones connected to at least one other clone in the repertoire at LD_1_, LD_≤2_, …, LD_≤12_ in pre-B cells, naïve B cells, plasma cells. **e** The power-law (orange), exponential (red) and Poisson (gray) distributions were fit to the cumulative degree distributions of naïve B cell and plasma cell (unimmunized) repertoires of a representative sample for similarity layers LD_1,3,7_ (log-log scale). Representative clusters are shown for LD_1_. **f** Percentage of CDR3 clones (mean ± s.e.m) that compose the maximal core. Subgraph of the maximal k-core (red), and k-1 (black), k-2 (dark gray) and k-3 (light gray) cores in a representative mouse pBC sample. **g** Percentage overlap of CDR3 germline V-genes in the maximal core of nBC repertoires (*n* = 5 mice and data sets for Unimm (unimmunized), OVA, NP-HEL, *n* = 4 mice sets for HBsAg, mean ± s.e.m). **h** Normalized neighborhood size for orders *n* = {1–10, 15, 20, 30, 40, 50} across CDR3 clones (similarity layer LD_1_). For a, b, d, barplots show mean ± s.e.m; for a–e, each B-cell stage *n* = 19 mice. Source data are provided as a Source Data file

In order to quantify the extent to which antibody repertoire architecture is reproducible across individuals, we analyzed the conservation of global (repertoire-level) network measures in the base similarity layer (similarity layer LD_1_). The base layer of the network organization provides information regarding the minimal differences (i.e., 1 a.a.) of all antibody sequences that compose the repertoire. While global network measures take into account all nodes (clones) of a network (Supplementary Table 1), local (clonal) network measures, discussed in the next section, are node-based (Supplementary Table 2). We used classical graph analysis parameters to characterize and quantify antibody repertoires from a systems’ prospective. Although antibody sequence diversity varied highly among mice (74–85% unique clones in a given mouse, Supplementary Fig. 2a), we found a remarkable cross-mouse consistency in clonal interconnectedness (similarity of antibody sequences) within each B-cell stage: the number of edges (*E*) among clones (*E*_pBC_ = 230,395 ± 23,048; *E*_nBC_ = 1,016,928 ± 67,080; *E*_*PC*_ = 45 ± 10), the size of the largest component (*pBC* = 46 ± 0.7%; *nBC* = 58 ± 0.5%; *PC* = 10 ± 1.6%; Fig. 2a) and cluster composition (Supplementary Fig. 2b) varied negligibly across mice (see Methods, Network analysis*)*. Thus, although antibody sequence composition varied substantially between individuals (Supplementary Fig. 2a), the overall structure of the network was similar indicating that the similarity relations of antibodies across B-cell stages and individuals were comparable. This finding suggests that VDJ recombination, although independent across individuals, generates antibody repertoires with convergent architecture.

Along B-cell development, PC repertoires were five-fold more disconnected than pBC and nBC networks (PC largest component was nearly five times smaller than pBC and nBC, Fig. 2a), and their centrality was concentrated on specific clones compared to the homogeneously connected clones in pBC and nBC networks (centralization *z*_*PC*_ = 0.05, density *D*_*PC*_ = 0.01, *z*_*pBC*,*nBC*_ ≈ *D*_*nPC*,*nBC*_ ≈ 0, Supplementary Fig. 2c). This result suggests that early in B-cell development, the architecture of sequence similarity covers a more continuous sequence space, while PC show a more heterogeneous antibody sequence composition. Compared to pBC, nBC showed a higher average degree (*k*_pBC_ = 3, *k*_nBC_ = 5, *k*_PC_ = 1, Supplementary Fig. 2b) although both repertoire compartments had identical diameter (*d*_pBC,nBC_ = 26,*d*_PC_ = 6, Fig. 2b), indicating a similar coverage of the sequence space. We observed that clones in pBC and nBC repertoires connected to comparable clones in terms of degree (assortativity^27–29^, *r*_pBC_ = 0.48, *r*_nBC_ = 0.41, Fig. 2b), whereas PC networks were consistently disassortative: their highly connected clones were linked to clones with few connections (*r*_PC_ = −0.09, Fig. 2b). The assortativity analysis may reflect the ‘praetorian’ nature of B-cell repertoires: prior to antigen exposure, all clones are equally important for antigen recognition, while showing the sequence bias after antigen-driven selection and expansion for plasma cells (complementary to clonal count). The characterization of the global patterns of antibody repertoire networks indicated that pBC, nBC and PC repertoires were reproducible. pBC and nBC clones cover a larger diversity space than clones in PC repertoires, where sequence similarity shows to be centralized and targeted towards certain clones.

### Clonal features of antibody networks are reproducible

Antibody repertoire architecture was also reproducible at the level of clonal (local, Supplementary Table 1 and Supplementary Table 2) features: pBC and nBC networks were characterized by a low variability (coefficient of variation as a measure of relative variability, CV) across various clonal parameters. The low variability of clonal parameters in pBC and nBC networks (*CV*_pBC_ = 2–28%, *CV*_nBC_ = 1–24%) was in contrast to the higher variability observed in PC repertoires (*CV*_PC_ = 13–118%, Fig. 2c). Specifically, low variability across different individuals was observed in several clonal parameters such as degree, transitivity, authority and PageRank, closeness and betweenness (Fig. 2c, Supplementary Fig. 2d). Variation analysis of the similarity degree indicated that the average number of similar clones to each of the clones in a repertoire varied marginally in pBC and nBC (*CV*_pBC,nBC_ = 5,6%). Transitivity showed that the similarity between clones both similar to a third CDR3 clone varied only negligibly between individuals (*CV*_pBC,nBC_ = 1,2%, Fig. 2c, Supplementary Fig. 2c). Authority and PageRank showed that the centrality of a CDR3 in the repertoire topology varied respectively *CV*_*pBC*,*nBC*_ = 11 and 25% across individuals, suggesting that individual repertoires were centered variably around certain CDR3 clones which were centers of highly connected (similar) clonal regions compared to less connected regions in the same repertoire network (Fig. 2c, Supplementary Fig. 2d). Closeness analysis revealed that an analogous number of similarity edges were required to access every other CDR3 from a given CDR3 clone in antibody repertoire networks of different individuals, as the similarity of a clone to every other CDR3 clone in the repertoire varied by *CV*_pBC,nBC_ = 17% (Fig. 2c, Supplementary Fig. 2d). Betweenness, the “bridge” function of a clone in sequence similarity, varied slightly across individuals with *CV*_pBC,nBC_ = 28% (Fig. 2c, Supplementary Fig. 2d), suggesting a comparable structure of the similarity route function of CDR3 sequences in these repertoires. Clones in pBC and nBC antibody repertoires cover a larger space and clonal similarity is homogenously distributed at the global repertoire level. Thus, clones of antibody repertoires in early B-cell development carry a similar centrality function within the architecture of the repertoire. These vary negligibly across individuals in their local network parameters, suggesting a homogeneous sequence-role among clones within and across repertoires.

Although a higher variability was detected across PC repertoire networks (Fig. 2c), clonal parameters were specific to B-cell stages (p_pBC,nBC/PC_ < 0.05): PC clones possessed higher centrality compared to pBC and nBC (closeness^30^, eigenvector^31,32^, and PageRank), while antigen-inexperienced clones showed to function as a bridge to sequence similarity (betweenness^33^, Supplementary Fig. 2d). Thus, antigen-experienced antibody clones differentiated in their centrality function; certain antibodies had a central position (high authority) in the architecture of the repertoire, with many similar antibodies. In contrast, early B-cell clones showed a connector function, bridging the sequence space of the repertoire.

Furthermore, in contrast to pBC and nBC, PC network clonal parameters correlated with CDR3 frequency (clonal degree median *r*_Pearson_ = 0.55, betweenness *r*_Pearson_ = 0.82) suggesting that clonally expanded CDR3 sequences were structural centers of similar clones (Supplementary Fig. 2e, f). This indicates that selection of highly frequent CDR3 clones within repertoires for antibody discovery might be a good proxy for selecting sequences that have a central role in the structure of sequence architecture, being centers of similarity. CDR3 authority correlated positively with germline V-gene frequency in PC clones (*r*_Pearson_ = 0.39), denoting the potential role of the V-gene usage in the centralization of these networks (Supplementary Fig. 2g). Thus, certain high frequency V-genes predispose clones to be highly connected and similar.

### The structure of antibody repertoires is reproducible

Network analysis revealed that antibody repertoires were constricted along B-cell development throughout all similarity layers. At LD_1_, 44–62% of clones were similar (connected) to at least one other clone in all B-cell stages, revealing a high sequence degeneracy in clonal generation and selection (Fig. 2d). This indicated that nearly half of the antibody sequences were similar to one another, thus demonstrating a high extent of repertoire constriction.

In order to understand if such degeneracy in CDR3 sequence similarity translated into reproducible repertoire network structures^31^, we determined the clonal empirical degree distribution. Degree distribution is a distinctive feature of different types of networks and it provides an immediate indication of how similarities (degrees) between antibody sequences are distributed in repertoires. Analysis of the cumulative degree distribution revealed that antigen-inexperienced pBC, nBC and unimmunized PC repertoires were exponentially distributed (LD_1_), whereas PC repertoires of immunized cohorts were power-law distributed (base similarity layer LD_1_, Fig. 2e, Supplementary Fig. 3d–g). Thus, the probability that antigen-inexperienced CDR3 clones were similar to one another was exponentially distributed, while the probability that antigen-experienced antibodies were similar to another one in the repertoire followed a power-law (scale-free for several samples but not all). Clusters of connected CDR3 clones showed a typical tree-like structure for pBC and nBC (generated by VDJ recombination/nucleotide additions/deletions), and a star-like structure for PC (likely generated by SHM). The structure of the network suggested an extended and chain-like sequence-similarity of the antibody clones for pBC and nBC repertoires, reflecting the vast sequence space that these repertoires need to cover in order to respond to the huge diversity of potential pathogens. The star-like structures of antigen-experienced repertoires suggests targeted expansion (one or few central clones that are similar to a large number of secondary clones) of certain antigen-responding PC clones after immunization.

In order to investigate if antibody repertoire network structures were reproducible across species, we constructed large-scale CDR3 networks with up to 6 million clones from human memory and naïve B-cell samples (Supplementary Table 3) and analyzed their degree distribution. The degree distribution of human memory B-cell CDR3 networks was exponential (Supplementary Fig. 4a). In line with what was already observed in murine samples, human naïve B-cell repertoires showed an exponential structure (Supplementary Fig. 4b). Thus, human B-cell repertoires networks were also structurally reproducible.

In order to prove the tree-/star-like hypothesis and further investigate the sequence similarity space, we performed *k*-core^34^ decomposition (where core is a “shell” englobing similar CDR3) and neighborhood analysis (Fig. 2f, g, h). A *k*-core decomposition was performed by iteratively removing *k* shells of all vertices of lower than a certain degree and leaving only the sequential cores of a network, its connected components. The *k*-core decomposition revealed that the largest *k*-cores (after all external shells with *k* < *k*_max_ were removed, where *k* is the degree, i.e., number of similar clones, see Methods) of pBC and nBC (0.04% and 0.06% of CDR3 clones in k-core, respectively) were 200-fold smaller than those of PC (8.2%, Fig. 2f). Antigen-inexperienced repertoires were thus characterized by larger coreness values (>20), signifying a more shell-like structure of CDR3 similarity (Supplementary Fig. 2j, k) and confirming their tree-like structure. Furthermore, the high convergence of V-genes at the core-level of antibody repertoire networks (pBC = 50%, nBC = 70%, PC = 1–10%, Fig. 2g), in contrast with the low exact CDR3 sequence core-overlap (Supplementary Fig. 3a, b, c), suggested a genetically determined origin of the structure. In naïve B-cell repertoires, 70% of V-genes represented in the core overlap between cohorts suggesting a starting bias focused on certain V-genes for the structure of the core architecture of CDR3 sequences.

The average CDR3 neighborhood size, which designated the set of similar CDR3 clones along each sequential step of similarity from a certain clone (orders *n* = 1–50), was order-independent in PC and plateaued at 2% of the network, confirming that PC clones were connected to one central clone in a star-like similarity structure, reflecting antigen-driven clonal selection and expansion. Neighborhood size^35^, the number of similar clones to each clone, increased with the order of the network in antigen-inexperienced cells up to 34% (Fig. 2h), signifying tree-like similarity structures that enable maximal exploration of sequence space within the genetically predetermined repertoire constriction space. This result suggests that antibody repertoires are evolutionarily wired to respond to diverse antigenic stimuli.

### Antibody repertoires are highly robust systems

We hypothesized that the reproducible architecture of antibody repertoires may have evolved to be robust to fluctuations in clonal composition. It is known that B cells and antibody repertoires are very dynamic systems characterized by a high turnover rate^36–38^. Therefore, we investigated the robustness of antibody repertoire architecture to clonal removal (deletion).

**Fig. 3:**
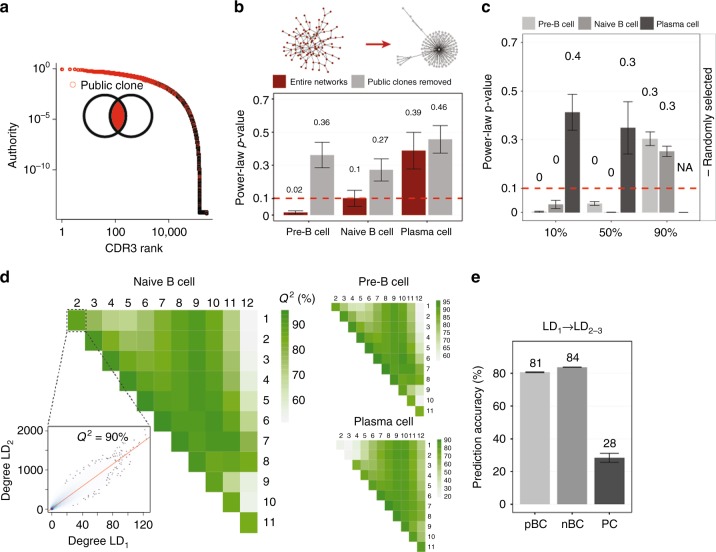
The architecture of antibody repertoires is robust and redundant. **a** CDR3 clones of an exemplary naïve B-cell repertoire (OVA-immunized mouse) have been ordered from increasing to decreasing frequency (CDR3 rank). Public clones are color-coded in red. **b** Bootstrapped *p*-values of the power-law fit are shown for complete antibody repertoires and after removing public clones. Power law is a good fit to degree distributions for p-values above the dashed red line (*p*-value = 0.1, Wilcoxon test). Examples of exponential (red) and power-law (gray) networks are shown on the top panel. **c** CDR3 clones were removed randomly at 10%, 50%, and 90% from each original repertoire (20 times) and the power-law distribution was fit to the cumulative degree distributions of the remaining CDR3 clones. A *p*-value = 0.1 (Wilcoxon test) is indicated as a red dashed line. In PC samples a fit was not feasible after removal of 90% of CDR3 clones (NA). **d** Heatmaps indicate the mean prediction accuracy (*Q*^*2*^, leave-one-out cross-validated *R*^*2*^) of similarity layer LD_1_ versus similarity layers LD_2–12_. The scatterplot shows *Q*^*2*^ for LD_1_ vs. LD_2_ for each CDR3 clone. **e** Prediction accuracy (*Q*^*2*^) for LD_1_ vs. LD_2_ and LD_3_. For b, c, e, barplots show mean ± s.e.m. Source data are provided as a Source Data file

It has been recently established that individual repertoires have public clones, which are defined as identical clones present in multiple individuals^12,39,40^. While mostly distinct, antibody repertoires still possessed a substantial fraction of public clones^39,41,42^ (15–26% along B-cell development, Supplementary Fig. 2a). Given their considerable proportion within a repertoire, we determined if public clones were essential to the maintenance of antibody repertoire architecture. We found that public clones ranged consistently among the highest authority clones (authority: degree of clonal connectedness, Supplementary Table 2), but were distributed across the entire authority range in antigen-inexperienced B-cell repertoires (Fig. 3a). Up to 74% of private clones (specific to an individual) were connected to at least one public clone (Supplementary Fig. 2i). To quantify the extent to which public clones maintain the architecture of antibody repertoires, we tested the effect of removing public clones on CDR3 degree distributions. In pBC and nBC, removal of all public clones transformed their network structure from exponential to power-law; in contrast, removal of public clones did not change the power-law network structure of PC repertoires (Fig. 3b). To assess if such a structural shift was specifically due to the deletion of public clones, we removed (repeatedly) random subsets of clones representing a similar fraction of public clones. The structure of antibody repertoires was shown to be robust along B-cell stages at up to 50% removal of random clones in mice (Fig. 2e) and up to 90% removal in a human naïve repertoire (Supplementary Fig. 4c). The same structural shift in repertoire structure caused by the deletion of public clones could only be replicated by removing 90% of random clones (Fig. 3c). Therefore, public clones represent pillars that are critical for maintaining the architecture of an antibody repertoire. The robustness of the antibody repertoire architecture suggests that functional immunity might be preserved even after extensive (random) loss or turnover of antibody clones (or B cells).

### Antibody repertoires are evolutionary redundant

Redundancy is a hallmark of robust systems; for example, redundancy in genes with the same function is the main mechanism of robustness against mutations in genetic networks^34^. To investigate the extent of redundancy within antibody networks, we examined whether their architecture at the base similarity layer (LD_1_) was manifested in higher order similarity layers (LD_>1_). Differences greater than one a.a. between antibody sequences could represent the potential personal scenarios of antibody repertoire evolution (somatic hypermutation^15,16^), a result of successful survival through selective processes. Specifically, if a clone connected to many other clones in the LD_1_ similarity layer mutates into a similar clone at a specific a.a. position, this *potential* clone will be connected to many clones in the LD_2_ similarity layer. Thus, higher order similarity layers can serve as surrogates for the evolution of potential antibody repertoires from antigen-inexperienced B-cell populations.

To quantify the extent of redundancy across similarity layers, we calculated the prediction accuracy of LD_1_ versus similarity layers LD_2–12_ using a leave-one-out cross-validation approach (Fig. 3d, Supplementary Fig. 3h, i). Specifically, quantitative redundancy was low in PC (LD_1_ → LD_2–3_ prediction accuracy was 28% on average); however, LD_1_ of pBC and nBC predicted CDR3 degree profiles of proximal similarity layers LD_2–3_ with ≥80% accuracy (Fig. 3d, e), thereby indicating a high redundancy in antibody repertoire architecture. This high redundancy is explained by the structure of the antibody networks (Fig. 2e–h). Although the distance between proximal similarity layers (LD_1_ to LD_3_) seems small (1–3 a.a. CDR3 sequence differences), it represents ≈20% of potential change in clonal a.a. sequence (99% of CDR3 clones are 4–20 a.a. long), which is in the range of highly mutated antibodies (e.g., broadly-neutralizing HIV-specific^43^). Therefore, redundancy in the antigen-inexperienced repertoire is maintained throughout a large sequence space and provides details on the pre-programmed evolvability^44,45^ of antibody responses.

## Discussion

Large-scale networks capture similarity relations between antibody clones that are not deductible from diversity analysis based clonal counts, thus providing an additional and complementary layer of information on the sequence diversity of a repertoire. Leveraging a custom-developed analysis platform for generating large-scale networks from datasets of millions of unique CDR3 a.a. sequences, we have discovered fundamental principles of antibody repertoire architecture such as: (i) reproducibility (ii) robustness and (iii) redundancy. We were able to detect a high cross-individual reproducibility by quantifying network parameters^27–29^ of antibody repertoires along B-cell development at the global (size, diameter and assortativity) and clonal levels (degree, transitivity, authority, closeness, betweenness, PageRank). Importantly, the reproducible clonal similarity structure was suggestive of the underlying immunobiology of each B-cell stage: human and murine antigen-inexperienced repertoires covered an extended sequence diversity space (tree-like exponential similarity structure) to counter high antigen diversity whereas, antigen-experienced repertoires presented a centralized network structure (star-like, power law), with many clones being similar to one central clone possibly originating from antigen-dependent clonal expansion and selection^47–49^. However, due to the smaller sample size of PC repertoires, this result should not be over interpreted for this B-cell stage. While counts of unique clones or clonotypes have been used so far as a dominating proxy for the diversity of antibody repertoires, network analysis introduces a novel and complementary layer of sequence diversity information. Networks can resolve the fine sequence structure of a repertoire or a synthetic (recombinant) antibody library; the breadth of a synthetic library may be assessed by statistically fitting its degree distribution to an underlying probability distribution. For example, power-law distributed synthetic libraries would be suboptimal for covering a large sequence space.

Large-scale network analysis of entire antibody repertoires revealed that these systems are robust enough to be amenable to subsampling, which is in contrast to other network systems^23,24^. Specifically, we showed that the structure of antibody repertoire networks was robust to extensive subsampling, with a removal of up to 50–90% of the clones. This result is crucial for the network analysis of human antibody repertoires, where biological subsampling remains an important problem^38,39^. While access to the entire human antibody repertoire is unfeasible, the robustness of the antibody repertoire sequence architecture to major subsampling shows that the structure of clonal sequence diversity is retained in even 10% of the original sample. This result is relevant for past and future BCR studies. The robustness of antibody repertoires might also explain their functionality despite large fluctuations of antibody repertoire composition over time^36–38^. Interestingly, the structure of murine antibody repertoires was fragile to the removal of public clones. The crucial role that public clones^12^ play as pillars of antibody repertoire architecture was revealed by large-scale networks, yet future research will need to determine the functional role (antigen specificity) of public clones in the humoral response.

We found that antibody repertoires presented intrinsic redundancy across similarity layers. This means that not only minimal differences (1 a.a. of the base layer LD_1_) but also further diversification (>1 a.a. differences between antibody sequences) may be hardcoded into the constricted sequence space of antibody repertoires, thus rendering their evolvability robust (analogously to other biological systems such as transcription factor networks^45^). The redundancy of antibody sequential similarities (LD > 1) might serve as a predictor of the immune response (development of certain sequences in time) following certain mutational pathways from the base layer of an individual antibody repertoire. This redundancy principle would potentially account only for the one-time snapshot of a repertoire and the high turnover rate of B-cells.

This work delineates guidelines for the large-scale network construction and analysis of large and diverse immune repertoires. In particular, our network analysis approach can be used where a partial biological coverage of the repertoire is available, although this might depend on the B-cell stage, species, and similarity layer investigated. The network quantitative analysis of global and clonal properties of adaptive immune repertoires (antibody and T-cell receptor repertoires) in health and disease is essential to comprehensively understand their architecture and may resolve limitations arising from visualization of graphics featuring high-dimensional data. It is of great interest to the field of immunological research to analyze disease-associated datasets in order to compare the architecture of healthy individuals versus a disease status and across diseases. However, in order for those studies to be of statistical, technological and immunological significance, novel sequencing efforts of large sorted B-cell populations are needed. As the field moves towards high-throughput single-cell analysis, future work might expand network analysis from clonal CDR3 sequence to clonotypes (e.g., through subgraph analysis), full length (VDJ) immune receptor sequences and paired sequences^7–12^.

The principles of the architecture of antibody repertoires uncovered here through network analysis may serve as a blueprint for the construction of synthetic antibody repertoires, which may be used to simulate natural humoral immunity for monoclonal antibody drug discovery and vaccine development^44,48^. Synthetic (recombinant) antibody libraries and their screening (e.g., by phage display) are highly utilized in antibody drug discovery^49^. However, it is also true that most antibody drugs have been isolated from natural antibody repertoires (e.g., in vivo selection from mice). Therefore, mirroring the network architecture of the natural antibody repertoire may be advantageous for improving the quality of synthetic antibody libraries and their screening for drug candidates. In order to achieve this, one may leverage network construction algorithms that specifically mirror exponential distributions in sequence space, thus producing synthetic libraries that recapitulate the diversity of naïve B cells. Furthermore, network analysis can serve to the identification of clones which lead to major alterations in repertoire composition^7^ and are responsible for repertoire transforming diseases such as autoimmunity or lymphomas^50,51^. The identification of these central clones in the network structure may allow for interventions to modify disease progression on the repertoire level by precision therapeutic clonal targeting^52^. Jardine and colleagues have shown that the targeted clonal expansion of selected B-cells is possible^52^. In the future, precise targeting of highly connected clones (e.g., public clones) may be useful also for therapeutic remodeling of network structure, if different disease stages are shown to be connected with specific network architectures. Last, we envision that large-scale antibody network analysis could be useful in personalized medicine in the prediction of immunity scenarios because of the redundancy that antibody repertoires present in their architecture. The architecture is just a snapshot of the repertoire at a given time. However, the intrinsic similarity relations among all clones as nodes in the networks can make potential sequence changes trackable and their probabilities may be assigned toward which sequence space might develop. In conclusion, we believe the stage is set for a rapid progression of the present guidelines into what was long ago envisioned by Niels K. Jerne^53^: the field of *network systems immunology*, which offers the potential to obtain greater understanding of the complexity of immune responses.

## Methods

### Mouse dataset

The dataset analyzed was produced as described by Greiff et al.^21^. Briefly, murine B-cell populations of pre-B cells (pBC, IgM, bone marrow, ≈7.5 × 10^5^ cells/mouse, c-kit^–^CD19^+^IgM^–^CD25^+^PI^–^), naïve follicular B cells (nBC, IgM, spleen, ≈1 × 10^6^ cells/mouse, CD138^–^CD19^+^IgD^2+^IgM^+^CD23^2+^CD21^+^PI^–^), and memory plasma cells (PC, IgG, bone marrow, ≈9 × 10^4^ cells/mouse, CD138^+^CD22^–^MHCII^–^CD19^–^IgM^–^PI^–^) were sorted using fluorescence-activated cell sorting (FACS) from C57BL/6 J mice unimmunized (*n* = 5) or prime-boost immunized with alum-precipitated antigens: nitrophenylacetyl-conjugated hen egg lysozyme (NP-HEL, *n* = 5), ovalbumin (OVA, *n* = 5) or Hepatitis B virus surface antigen (HBsAg, *n* = 4). Following total RNA extraction, full-length antibody variable heavy chain (VDJ) libraries were generated by a two-step PCR process, as described previously^54^. Libraries were sequenced using the Illumina MiSeq (2 × 300 bp) platform. Mean Phred-scores of raw data were ≥30. Approximate paired-end reads (full-length VDJ) were: pBC ≈ 5 × 10^6^ reads (untreated *n* = 1,666,407, NP-HEL *n* = 2,306,769, OVA *n* = 2,337,876 and HBsAg *n* = 2 330 505 sequencing reads), nBC ≈ 10 × 10^6^ reads (untreated *n* = 6 487 616, NP-HEL *n* = 4 157 887, OVA *n* = 4 245 486 and HBsAg *n* = 6,076,876 sequencing reads) and PC ≈ 4 × 10^6^ reads (untreated *n* = 188 440, NP-HEL *n* = 125 118, OVA *n* = 194,003, and HBsAg *n* = 121,382 sequencing reads)^21^. The experimental design of the study minimized technological (sequence undersampling) and biological undersampling (cell undersampling) as explained in depth in a previous publication^21^.

### Data preprocessing and CDR3 clonal analysis

Antibody read sequences have been preprocessed and VDJ annotated with MiXCR^55^ and further filtered to retain only those sequences that had CDR3 length ≥4 a.a. and occurred more than once in each CDR3 repertoire data set (Supplementary Fig. 1a). Clones were defined by 100% a.a. sequence identity of CDR3 regions. CDR3 regions were defined by MiXCR according to the nomenclature of the Immunogenetics Database (IMGT)^56^. Unique mean CDR3 a.a. clones analyzed for pBC cohorts were untreated *n* = 152,859, NP-HEL *n* = 185,128, OVA *n* = 188,971 and HBsAg *n* = 159,546; nBC untreated *n* = 424 940, NP-HEL *n* = 395 048, OVA *n* = 330 466 and HBsAg *n* = 440 834; PC untreated *n* = 143, NP-HEL *n* = 156, OVA *n* = 154 and HBsAg *n* = 132.

### Human dataset

Sequencing data of naïve and memory B cells from three healthy human donors were published by DeWitt et al.^20^ and downloaded already preprocessed from http://datadryad.org/resource/doi:10.5061/dryad.35ks2. The dataset contains 2–4 × 10^7^ naïve B-cells and 1.5–2 × 10^7^ memory B-cells for each donor. Unique CDR3 a.a. clones analyzed were D1-M *n* = 2,305,669, D2-M *n* = 1,836,019, D3-M *n* = 3,127,059 for memory B cells and D1-Na *n* = 6 187 146, D1-Nb *n* = 5,716,124, D2-N *n* = 4,408,661, and D3-N *n* = 6,348,502 for naïve B cells (Supplementary Table 3). After alignment and preprocessing, we constructed large-scale networks from unique CDR3 of ≈6 million nodes.

### Network construction

To construct networks (graphs), a sparse triangle matrix of pairwise Levenshtein distances (LD) between CDR3s must first be computed. For small samples (up to 100,000 unique CDR3 sequences) such a calculation is relatively fast on a single computer. However, due to the N^2^ complexity of required calculations, computing the pairwise matrix for samples of >100,000 unique CDR3 sequences becomes prohibitively expensive. To perform these computations, we developed software that utilizes the Apache Spark (*2*) distributed computing framework to partition the work among a cluster of many machines (Supplementary Fig. 1b). We chose specifically Apache Spark because (i) its deployment is very flexible with regard to underlying computing infrastructure and (ii) for similarity layers LD_>1_, the networks become extremely large and difficult to process. When two sequences were similar within a defined threshold (Levenshtein distance, LD = 1–12), they were connected in the repertoire network (i.e., similarities of 1 a.a. differences were captured in similarity layer 1, LD_1_, 2 a.a. in LD_2_ and so on). In these cases, our package can take advantage of the Spark Graph Frames distributed graph library^57^, which allows scaling to even larger samples with millions of sequences (Supplementary Fig. 1c). With this approach we were able to compute the distance matrices for large samples (>100,000 unique CDR3 sequences) within minutes (Supplementary Fig. 1b. c).

In addition to the computational complexity inherent in creating the distance matrix, the construction of networks for large LD is computationally expensive. We therefore avoided constructing networks altogether for calculating the node degrees and instead used a map-reduce distributed algorithm. For practical purposes, the construction of small networks was performed using the Networkx library^58,59^. For generating and outputting the largest graphs to disk in common network formats, we used the efficient graph-tool library (https://graph-tool.skewed.de/). For manipulating and analyzing the largest networks, our software package took advantage of the Spark GraphFrames distributed graph library as mentioned above^57^.

The software was developed in python (https://www.python.org/) using the Numpy/Scipy^60^ scientific libraries for matrix and array manipulation and Apache Spark^17^ as the distributed backend. Our software package for antibody repertoires *imNet* is available (https://github.com/rokroskar/imnet) and includes tutorials and demos, including scripts to set up the distributed computation environment on commonly-used compute cluster infrastructure The results shown in this work were obtained using 1–625 cores of the *Euler* parallel-computing cluster operated by ETH Zürich. In addition, imNet is a python library and can be used locally to work with both python 2 and 3.

### Degree distribution fits

Degrees (number of similar CDR3 sequences to a specific CDR3 sequence) were calculated for each of the similarity layers LD_1–12_ for each CDR3 sequence in each sample. CDR3 with zero degrees that were not similar to any other CDR3 in the network were excluded in order to fit degree distributions. The power-law, exponential and Poisson distributions were fitted to the empirical degree distributions of the networks, constructed as described in *Network construction*, by estimating *x*_min_ (estimated lower degree threshold by minimizing the Kolmogorov-Smirnoff statistic^61^) and optimizing model parameters using the poweRlaw^62^ package. We first discriminated if the power-law distribution could describe the best fit to the degree distribution by bootstrapping 100 times the power-law *p*-value obtained from each sample after estimating *x*_min_. Following the approach described by Virkar and Clauset^63^, a *p*-value ≥ 0.1 indicated that the power-law distribution described the degree distribution (Supplementary Fig. 1a). To determine the degree distribution in cases where the power law was not the best distribution fit (*p*-value < 0.1), we compared the exponential and the Poisson fits. Two-sided *p*-value ≈ 0 indicated that the fitted models could be discriminated, and one-sided *p*-value ≈ 1 (Wilcoxon test) indicated that the first (for example exponential) model was the best fit for the data^62^.

### Robustness

Public clones were defined as clones shared among at least two subjects in a cohort (Supplementary Fig. 2). In order to assess the robustness of the architecture of antibody repertoire networks, we removed public clones from each sample. As controls, we performed repeated removal (20 times) of randomly selected clones in the size of public clones. The *p*-values (Wilcoxon test) for the power-law fit were calculated after 100x bootstrapping for each repertoire; one-sided and two-sided *p*-values were used for the comparison between the exponential and the Poisson fits (see *Degree distribution fits*).

### Network analysis

Drawing from network theory^64^, we translated the concepts of network analysis^23^ to antibody repertoires. An antibody repertoire network is an undirected *graph* G = (V, E) described as a set of *nodes* (CDR3 vertices, V) together with a set of connections (similarity edges, E), representing the adjacency matrix A of pairwise Levenshtein distances (LD) between CDR3 a.a.

$${\mathrm{Sequences}}\,{\mathrm{A}} = \left( {\begin{array}{*{20}{c}} 0 & \cdots & {{\mathrm{LD}}_{1{\mathrm{n}}}} \\ \vdots & \ddots & \vdots \\ {{\mathrm{LD}}_{{\mathrm{n}}1}} & \cdots & {{\mathrm{LD}}_{{\mathrm{nn}}}} \end{array}} \right).$$

In the context of antibody repertoires, we let N = |V| and L = |E|. The *order* of a graph N represents the number of its unique CDR3 clones (nodes). The *size* of a graph L is the number of its CDR3 similarity connections (edges). The degree *k*, that represents the edges connected to a node, describes the count of all similar CDR3 clones to a CDR3 based on LD. Because the *degree* indicates how active a node is, it could be interpreted as a measure of how central a CDR3 clone is in the antibody repertoire network. In simpler terms, it quantifies the number of CDR3 clones that are similar to a certain CDR3, and thus the potential development or the evolutionary routes to this CDR3.

The *average degree* $\left\langle k \right\rangle \equiv \frac{{\mathop {\sum }\nolimits_{{\mathrm{i}} = 0}^{\mathrm{n}} {\mathrm{k}}_{\mathrm{i}}}}{{\mathrm{N}}} = \frac{{2{\mathrm{L}}}}{{\mathrm{N}}}$ is the average number of similar CDR3 clones. The *degree distribution* P(k) = N_k_/N, defined as the fraction of nodes with degree *k* (N_k_) in total nodes, represents the fraction of CDR3 clones that have the same number of similar CDR3s. The *cumulative degree distribution* ${\mathrm{P}}_{\mathrm{k}} = \mathop {\sum}\nolimits_{{\mathrm{k}}\prime = {\mathrm{k}}}^\infty {{\mathrm{p}}_{{\mathrm{k}}\prime }}$ describes the fraction of nodes with degree greater than or equal to *k*'. In Erdős–Rényi (ER) random graph models, degrees follow a Poisson distribution ${\mathrm{P}}\left( {\mathrm{k}} \right) = \frac{{\langle {\mathrm{k}}\rangle ^{\mathrm{k}}{\mathrm{e}}^{ - \langle {\mathrm{k}}\rangle }}}{{{\mathrm{k}}!}}$ in the limit of large numbers of nodes, while degree distributions have an exponential tail P(k) ~ e^−αk^ in exponential networks^65^.

Global characterization^23^ described the network as a whole, such as degree distribution, centralization, largest component, diameter, clustering coefficient, assortativity and coreness. The *centralization* analysis indicates if the network is homogeneous (clones are connected in the same way) or is centered around certain nodes (highly connected clonal regions compared to less connected regions in the same network). The *largest component* is the largest cluster of connected CDR3 clones. The *diameter (d)* is the maximum distance (shortest path between two nodes) between any pair of CDR3 sequences. The *clustering coefficient (C)* represents the probability that neighbors of a node are also connected, which translates in antibody repertoires as the probability that CDR3 clones similar to a specific CDR3 are also similar among one another. Network *density (D)* is the ratio of the number of edges (CDR3 similarities) and the number of all possible edges in the network. The *assortativity coefficient*^25^ *(r)* indicates if nodes in a network connect to nodes with similar characteristics. It is positive if nodes tend to connect to nodes that are similar to them (i.e., highly connected CDR3 sequences are similar and connect to highly connected CDR3 sequences), and negative otherwise. *Coreness* is a measure of the network’s cohesion and allows one to understand the global network structure and is useful in comparing complex networks by analyzing the subsets of CDR3-cores that form layers in the antibody repertoire. *K*-core decomposition is a process that is performed by iteratively removing shells of all vertices of degree less than *k* (*k* < *k*_max_) leaving the *k*-cores of a network (its connected component). The *k*-core of a graph is the maximal subgraph in which each node has at least degree *k*. We have computed the maximal *k*-core of antibody repertoire networks (the innermost core, *k*_max_) and the core distribution along *k* degrees.

Clonal (local) characterization of antibody repertoires was performed by analyzing local properties of the networks^23^. The importance of CDR3 clones was measured by calculating the authority^66^, eigenvector^27^ and PageRank^28^ scores of each node in repertoire networks. In particular, the *authority (a)* of nodes is defined as the principal eigenvector of the transpose matrix t(A)*A, where A is the adjacency matrix of the network. Eigenvector centrality indicates the centrality of a CDR3 clone, not only dependent on the number of similar CDR3 (number of degrees, connections) but also on the quality of those connections: CDR3-nodes with high eigenvector values are connected to many other nodes which are, in turn, connected to many others (and so on). *PageRank* measures the importance of the similarity between two CDR3 clones within the network extending beyond the approximation of a CDR3 importance or quality. *Closeness (centrality*^26^) *(c)* was calculated to measure how many steps were required to access every other CDR3 from a given CDR3 clone in antibody repertoire networks. We calculated the normalized closeness by multiplying the raw closeness by *n*-1, where *n* was the number of nodes in the network. *Clique* analysis identified maximally-connected subgraphs (a subset of nodes) in which every CDR3 was similar to every other CDR3 sequence and the largest clique was the maximal completed subgraph which had more nodes than any other clique in the network. The node betweenness (*b*) is the number of geodesics (shortest paths) going through a node and indicates the “bridge” function of a CDR3 sequence. Network properties were calculated using the igraph^67^ R package.

### Network properties

Units are numeric and dimensionless:

Network size is represented by the number of nodes (vertices) and/or number of edges (links, connections, degree).

Largest component size is the number of nodes in the largest component, calculated as the subgraph in which any two vertices are connected.

Diameter (numeric) is the largest number of vertices which must be traversed in order to travel from one vertex to another and is calculated by using a breadth-first search like method.

Assortativity coefficient^25^ r is a preference for a network’s nodes to attach to others that are similar in some way, e.g., the tendency of the nodes to connect with other nodes with similar degree values. The assortativity coefficient (*r*) is the Pearson correlation coefficient of the degrees at either ends of an edge and lies in the range −1 ≤ r ≤ 1: $r = \frac{1}{{\sigma _q^2}}\mathop {\sum }\limits_{jk} jk(e_{jk} - q_jq_k)$ where *e*_*jk*_ is the joint probability distribution of the remaining degrees of the two vertices at either end of a randomly chosen edge, symmetric in its indices on an undirected graph *e*_*jk*_ = *e*_*kj*_ obeying the rules (i) $\mathop {\sum}\nolimits_{jk} {e_{jk} = 1}$ and (ii) $\mathop {\sum}\nolimits_j {e_{jk} = q_k}$ (given that *p*_*k*_ is the probability that a randomly chosen node on the graph will have degree *k* and *q*_*k*_ is the normalized distribution of the remaining degree—the number of edges leaving the node other than the one selected $q_k = \frac{{(k + 1)_{p_{k + 1}}}}{{\mathop {\sum }\nolimits_j pj_j}}$). $\sigma _q^2$ is the variance (standard deviation) of the distribution *q*_*k*_ and it is useful when comparing networks in order to normalize.

Clusters are connected components of a graph. The cluster size is the number of connected nodes in a cluster. The cluster number is the number of clusters in a graph.

Clustering coefficient/transitivity^28,67^ is the the ratio of the triangles and the connected triples in an undirected graph. Let *e*_*j*_(*j*) denote the number of edges that connect the immediate neighbors of a node *j* and let *k*_*j*_ denote the node degree of *j*, that is, its number of immediate neighbors, the clustering coefficient is $C_j = \frac{{2 \ast e_j(j)}}{{k_j(k_j - 1)}}$. The clustering coefficient for the whole graph is the average of the local values: $C = \frac{1}{n}\mathop {\sum}\nolimits_{j = 1}^n {C_j}$

Density is the ratio of the number of edges and the number of possible edges.

Centralization is the network centrality indices which characterize each vertex/edge with respect to their position within the network.

Average degree is the average number of connected vertices.

Neighborhood of a vertex *v* is the number of vertices adjacent to *v*, the subgraph induced by all vertices adjacent to *v*: $N\left( G \right) = \mathop {\bigcup}\nolimits_{v \in G} {N(v)}$

Centrality measures the influence of a node in a network:

Eigenvector centrality score is the values of the first eigenvector of the graph adjacency matrix; the score is the result of a process in which the centrality of each vertex is proportional to the sum of the centralities of those vertices to which it is connected. In general, vertices with high eigenvector centralities are the ones connected to many other vertices which are, in turn, connected to many others and so on^67^: $x_v = \frac{1}{\lambda }\mathop {\sum }\limits_{t \in M(v)} x_t$ where *M*(*v*) is a set of the neighbors of and *λ* is a constant.

Authority is the centrality of each vertex proportional to the sum of the centralities of those connected to it.^67^ *a* = *t*(*A*)*A where *A* is the adjacency of the graph.

PageRank is a technique that identifies important nodes based on the link structure of the graph. Every node of the graph (*v*) is represented by a numerical score between 0 and 1, known as its *PageRank*^28^, *π*(*v*), which depends on the structure of the graph, i.e., the probability to reach any node from a given node, and on the value of *α* that expresses the teleport operation probability to jump from a node to any other node in the graph (fixed parameter chosen in advance). PageRank is the principal left eigenvector of the transition probability matrix *P* = *NxN*, characterizing a Markov chain of *N *states, where *P*_*ij*_ is the probability that the state at the next time-step is *j*, conditioned on the current state *i*. The left eigenvectors of the transition probability matrix *P* are *N*-vectors $\vec \pi$ such that $\vec \pi P = \lambda \vec \pi$. The $N$ entries in the principal eigenvector $\vec \pi$ are the PageRank values for the corresponding nodes.

Closeness centrality of a vertex measures how easily other vertices can be reached from it. It is defined as $C\left( v \right) = \frac{1}{{\mathop {\sum }\nolimits_w d(v,w)}}$ where *d*(*v*, *w*) is the distance between vertices *v* and *w*.

Betweenness centrality for each vertex is the number of the shortest paths that pass through it defined as $B\left( v \right) = \mathop {\sum}\nolimits_{s \ne v \ne t} {\frac{{\sigma _{st}(v)}}{{\sigma _{st}}}}$ where *σ*_st_ is the total number of shortest paths from node *s* to node *t* and *σ*_*st*_(*v*) is the number of those paths that pass through *v*.

### Quantifying the predictive performance (*Q*^2^) of linear regression models

The predictive performance (*Q*^2^) of each linear regression model (*Y* = *Xβ* + *ε*) was calculated using leave-one-out cross-validation (LOOCV): $Q^2 = \left( {1 - \frac{{{\mathrm{PRESS}}}}{{{\mathrm{TSS}}}}} \right) \cdot 100$, where PRESS is the predictive error sum of squares $\left( {\mathop {\sum}\nolimits_{j = 1}^n {\left( {Y_j - \hat Y_{[j]}} \right)^2} } \right.$ with $\hat Y_{[j]}$ denoting the prediction of the model when the *j*-th case is deleted from the training set and TSS is the total sum of squares $\left( {\mathop {\sum}\nolimits_{i = 1}^n {\left( {Y_j - \bar Y} \right)^2} } \right)$ (Greiff et al., 2012). *X* and *Y* are CDR3 degree vectors of repertoires at each LD_1–12_. LOOCV was performed using the forecast R package^68^. Cross-validation was used because, in contrast to regular regression analysis, it enables the quantification of the predictive performance of each regression model.

### Simulated networks

Networks (nodes *V* = 10^2^–10^5^) were simulated with the ER, exponential and power-law models using base R^69^ and igraph^67^. Random networks were simulated according to the ER model, exponential networks were simulated setting a probability of a connection between two nodes *p* = 0.5 and scale-free networks were simulated using the Barabási-Albert model (Barabási and Albert, 1999).

### Graphics

Graphic representations were produced using base R^69^ and the ggplot2 R package^70^. Heatmaps were produced using the NMF package^71^. Networks and network clusters visualization were performed using igraph^67^ employing the Fruchterman–Reingold force-directed and Kamada–Kawai layout algorithms. Large-scale networks (Fig. 1a) were visualized using Gephi (version 0.9.1)^72^; node size was scaled 10–100 proportional to the degree of a node and a blue to gray color gradient was applied to nodes from high to low degrees.

### Statistical significance

Statistical significance was tested using the Wilcoxon rank-sum test. Results were considered significant for *p* < 0.05.

### Reporting summary

Further information on experimental design is available in the Nature Research Reporting Summary linked to this article.

## Supplementary information

Supplementary Information

Reporting Summary

Source Data

## Data Availability

Software is available at https://github.com/rokroskar/imnet.

## Data availability

Antibody repertoire sequencing data analyzed is available with ArrayExpress accession number: E-MTAB-5349.
