# Supplementary material for: Large-scale network analysis reveals the sequence space architecture of antibody repertoires
Source: Nat Commun. 2019 Mar 21;10:1321. doi: 10.1038/s41467-019-09278-8 (PMC6428871; doi:10.1038/s41467-019-09278-8)
Supplement: Supplementary file 1 — Supplementary Information [file 41467_2019_9278_MOESM1_ESM.pdf]

SUPPLEMENTARY INFORMATION

Large-scale network analysis reveals the sequence space architecture of antibody repertoires

Miho et al.

Supplementary Figure 1

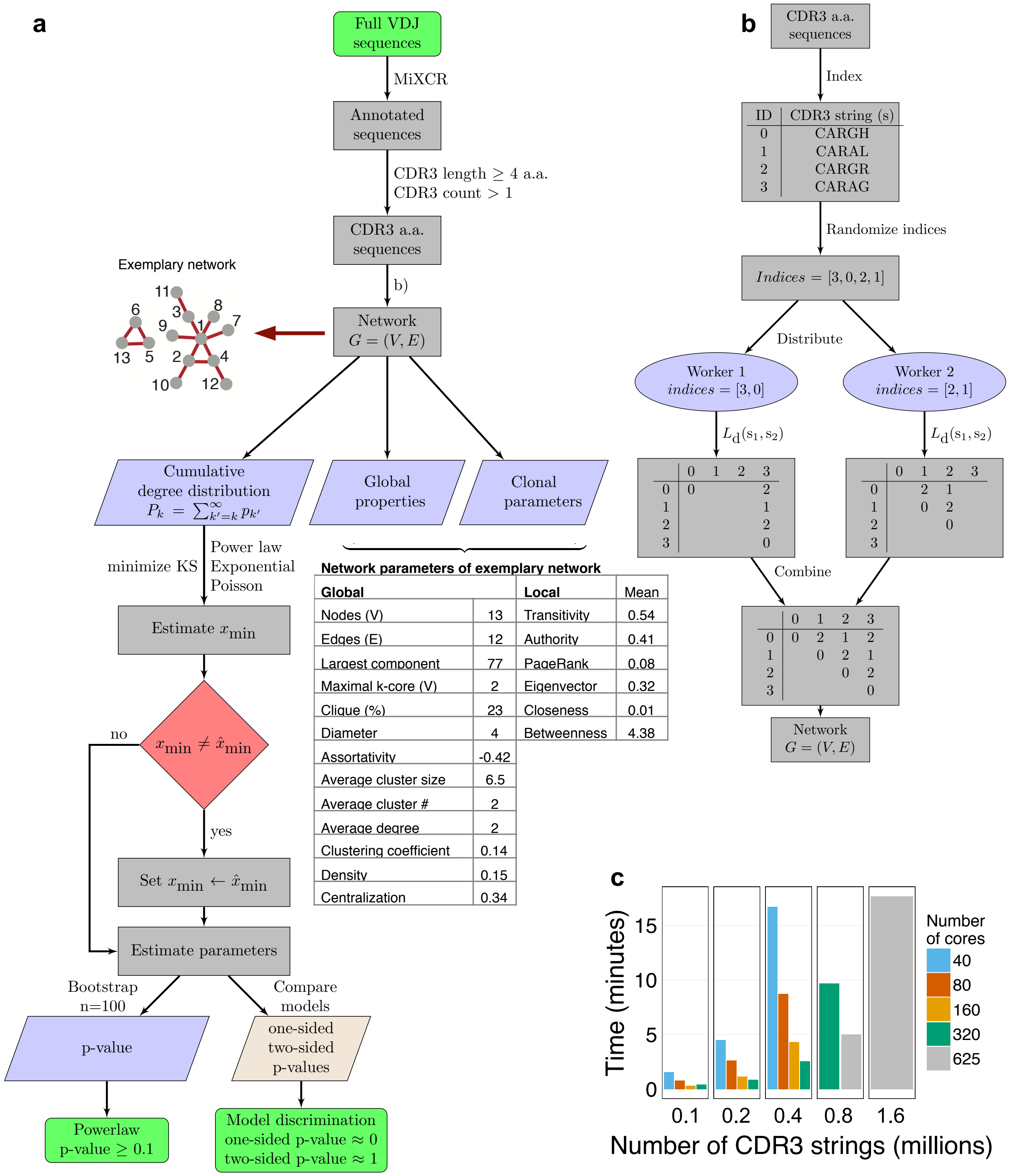

Network parameters of exemplary network

**Global**		**Local**	**Mean**
Nodes (V)	13	Transitivity	0.54
Edges (E)	12	Authority	0.41
Largest component	77	PageRank	0.08
Maximal k-core (V)	2	Eigenvector	0.32
Clique (%)	23	Closeness	0.01
Diameter	4	Betweenness	4.38
Assortativity	-0.42		
Average cluster size	6.5		
Average cluster #	2		
Average degree	2		
Clustering coefficient	0.14		
Density	0.15		
Centralization	0.34		

Supplementary Figure 1. High-performance computing platform to construct and analyze large-scale networks from entire antibody repertoires. (**a**) Data preprocessing, network construction and model fits to degree distribution (see Methods, Degree distribution fits for further details). Network parameters (global and mean local/clonal) are shown for the exemplary network. (**b**) Software schematics showing the distributed parallel computing platform used to partition the work among a cluster of many workers. (**c**) Computation time to construct large-scale networks depends on the number of CDR3 sequences and the number of cores used.

Supplementary Figure 2

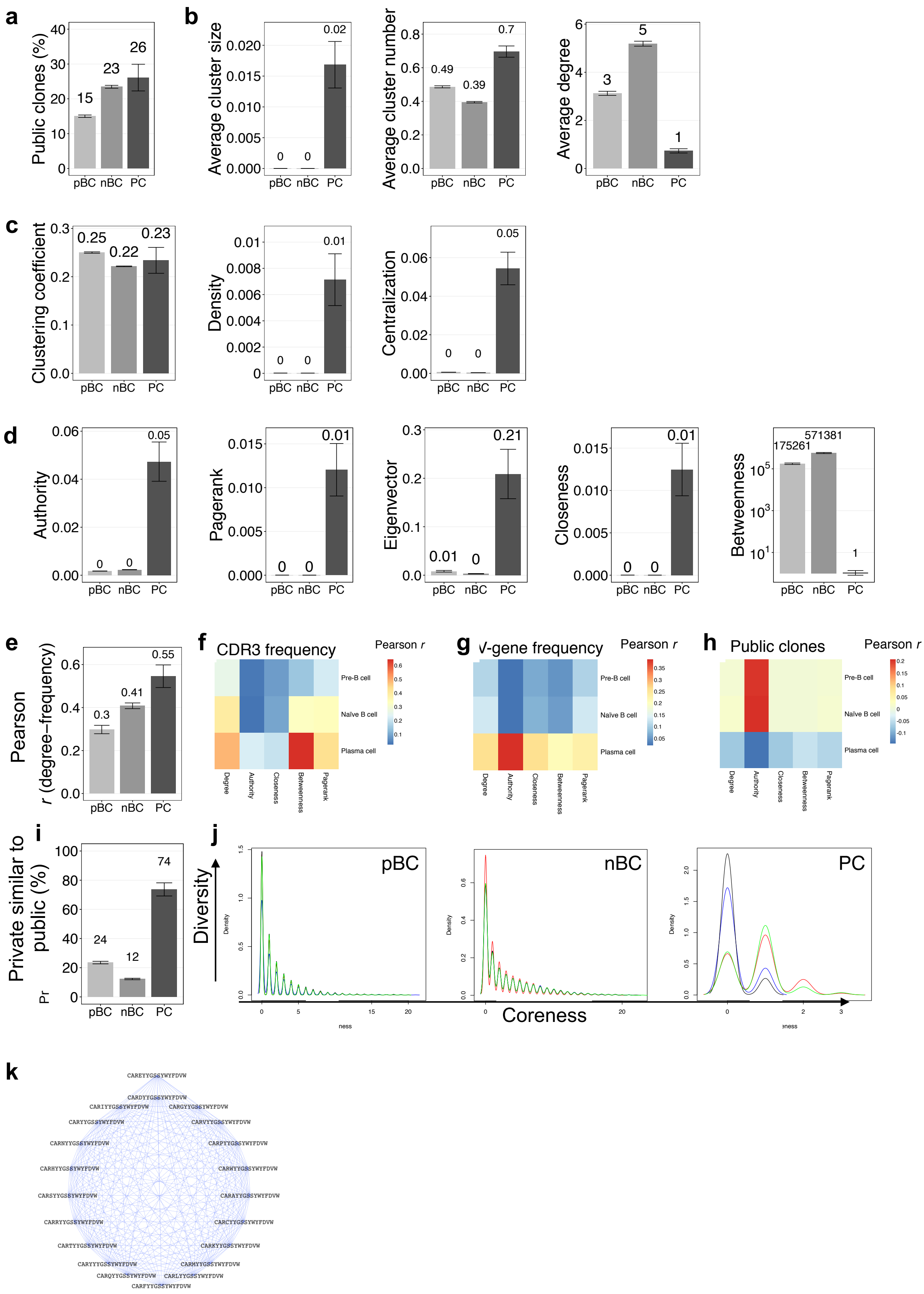

Supplementary Figure 2. Global and clonal (local) network parameters of antibody repertoires of pre-B cells (pBC), naïve B cells (nBC) and plasma cells (PC).

(**a**) Percentage of public clones, shared CDR3 clones between mice in pre-B cell (pBC), naïve B cell (nBC) and plasma cell (PC) repertoires.

(**b–c**) Global properties: Cluster analysis shows the average normalized cluster size and cluster number in the antibody repertoire networks (the resulting zero for pBC and nBC is due to rounding to the second significant digit <0.005). Average degree, clustering coefficient, density and (degree) centralization characterize the networks at the global level.

(**d**) Local properties: authority, PageRank, eigenvector, closeness and betweenness describe each clone in the network. Average values are shown for each B cell population, pre-B cells (pBC), naïve B cells (nBC) and plasma cells (PC). Barplots show mean±s.e.m, mice n=19.

(**e**) Pairwise Pearson correlation (*r*, mean±s.e.m) of CDR3 degree with CDR3 frequency in pre-B cells (pBC), naïve B cells (nBC) and plasma cells (PC) antibody repertoire networks.

(**f**) Pairwise Pearson correlation of local properties with CDR3 frequency (median, mice n=19).

(**g**) Pairwise Pearson correlation of local properties with germline V-gene frequency (mean, mice n=19).

(**h**) Pairwise Pearson correlation of CDR3 clonal (local) properties with public (1) vs. non-public (0) CDR3 clones (mean, mice n=19).

(**i**) Percentage of public clones similar (connected) to at least one other public CDR3 clone sequence by cohort (mean, mice n=19).

(**j**) Coreness density distribution for the unimmunized cohort of pre-B cells (pBC), naïve B cells (nBC) and plasma cells (PC). The x-axis shows the k-core after removing sequentially shells of nodes of degree k-1 ($max_{pBC/nBC}$20, max_{PC} 3). Line colors depict different mice.

(**k**) Example of CDR3 clones in the largest clique (complete connected subgraph) from a pre-B cell repertoire (NP-HEL cohort).

For Supplementary Figure 2a–e, i, barplots represent mean±s.e.m; for each B-cell stage, n=19 mice. Numbers have been rounded to the second decimal digit.

Supplementary Figure 3

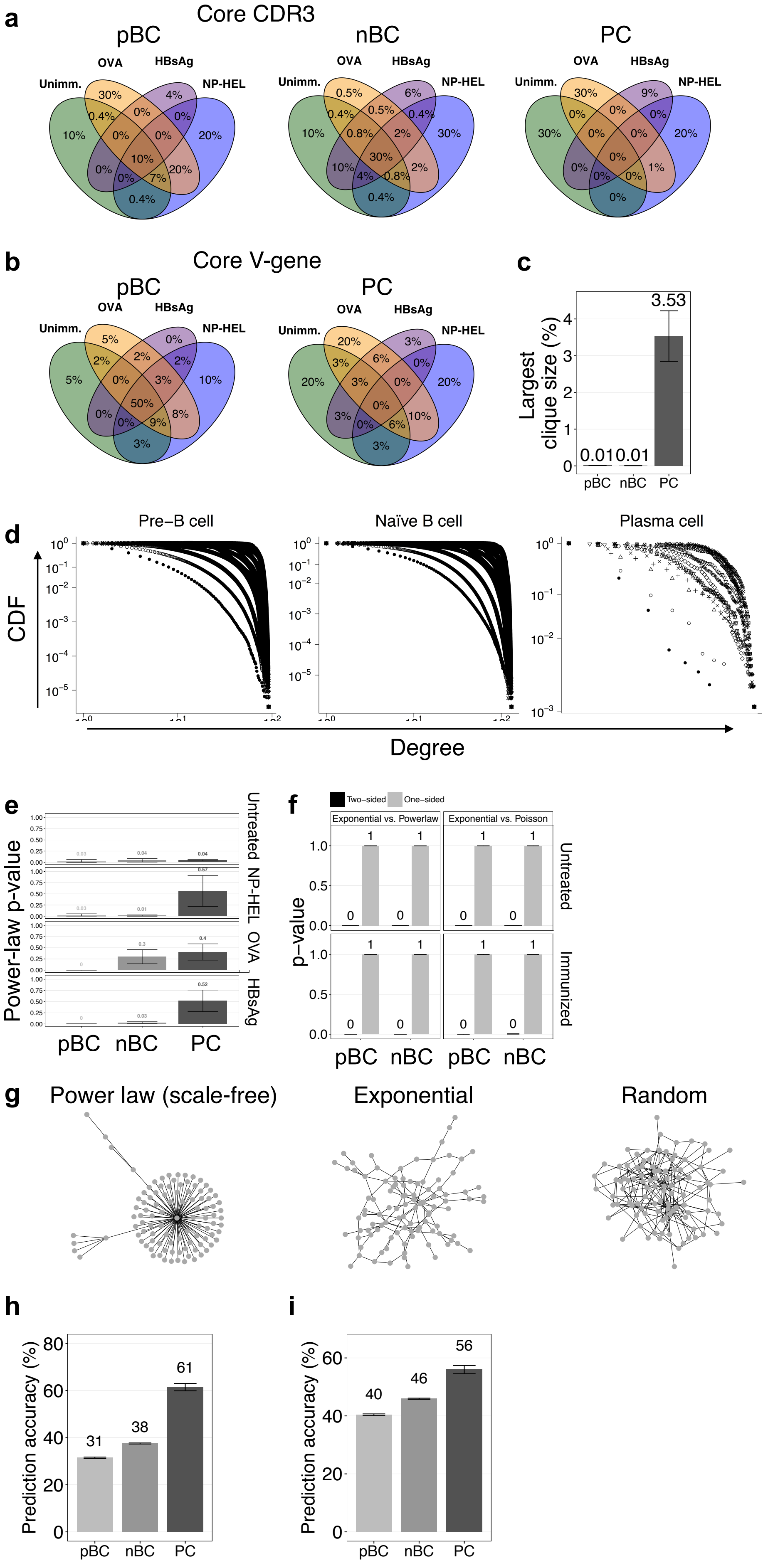

Supplementary Figure 3. Core and structure (degree distributions of CDR3 similarity) analysis, and similarity layer prediction of antibody repertoire networks.

(**a**) Maximal core CDR3 clones overlap in pre-B cells (pBC), naïve B cells (nBC) and plasma cell (PC) repertoire networks.

(**b**) Maximal core germline V-genes overlap in pre-B cells and plasma cell.

(**c**) Percentage of the largest cliques (completely connected subgraph) along B cell development. Barplots represent mean±s.e.m; for each B-cell stage, n=19 mice.

(**d**) Cumulative degree distributions (CDF). Each distribution line (different symbols) depicts one similarity layer LD_{1-12} (HBsAg-immunized mouse n. 4).

(**e**) p-values (Wilcoxon test, mean±s.e.m) of the power-law fit for each cohort.

(**f**) One-sided and two-sided p-values (Wilcoxon test, mean±s.e.m) for the discrimination between the exponential (one-sided p-value=1, two-sided p-value=0, Wilcoxon test) and the power-law fits.

(**g**) Graphics of power-law (α=2.2), exponential and random network models of 100 nodes.

(**h**) Prediction accuracy (Q^2, leave-one-out cross-validated R^2, mean±s.e.m) of selected distant similarity layers LD_{4-12} from LD_1.

(**i**) Prediction accuracy (Q^2, mean±s.e.m) of all similarity layers (LD_{2-12}) from LD_1.

Supplementary Figure 4

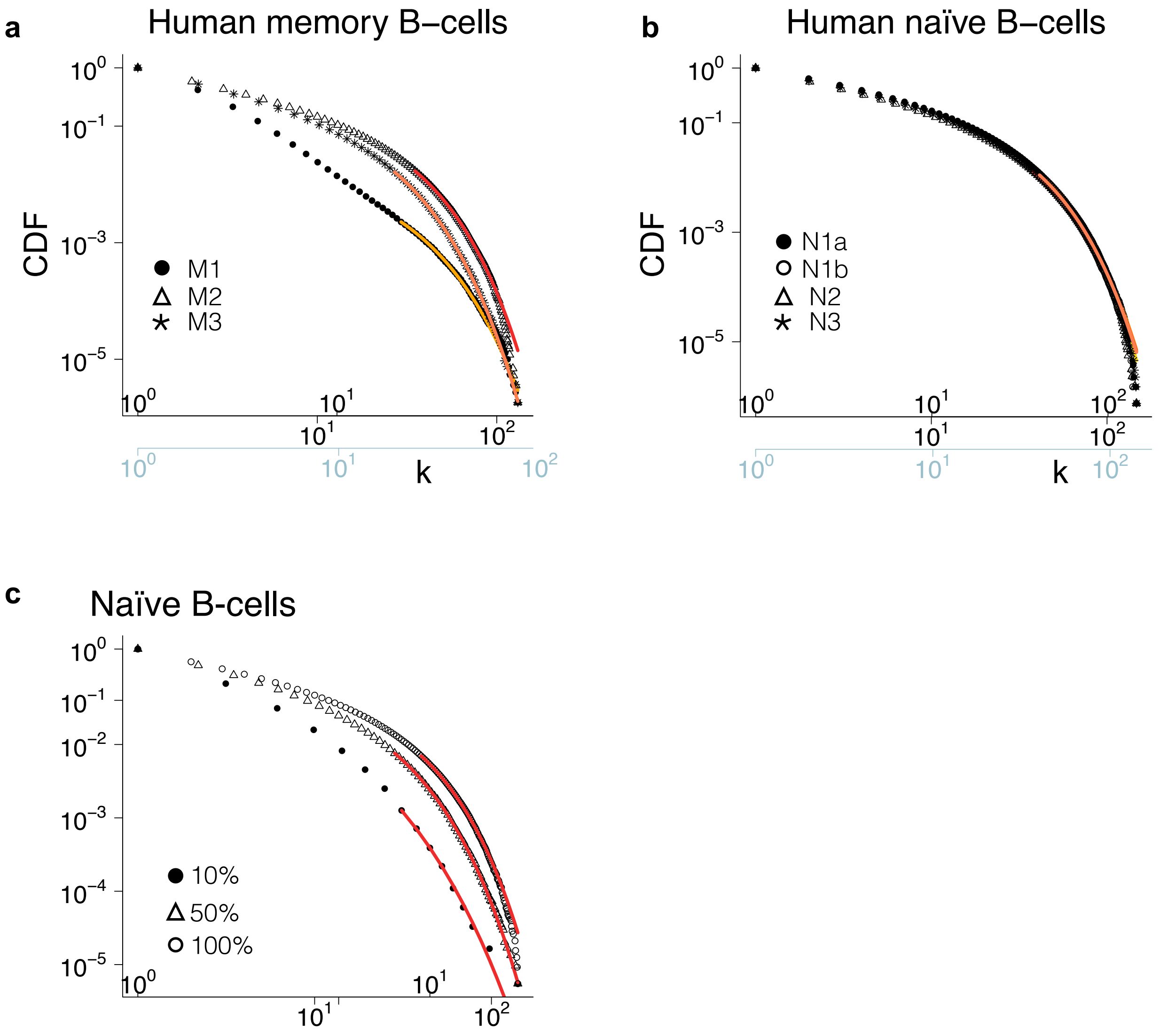

Supplementary Figure 4. Human memory and naïve B cell network structure.

(**a**) Degree distributions of large-scale human memory B-cell networks with exponential fits. Each individual M1, M2 and M3 samples are represented from different symbols. X-axis shows the degree (k) for each of the samples in order 1 to 3 from top to bottom.

(**b**) Degree distributions of large-scale human naïve B-cell networks with exponential fits. Each sample N1a and N1b (technical replicates), N2 and N3 is represented from different symbols. X-axis shows the degree (k) for each of the individuals in order 1 to 3 from top to bottom (one x-axis is shown for technical replicates).

(**c**) Degree distributions of subsampled human naïve B-cell networks. Different symbols show the degree distribution of when subsampling (random removal of clones) for 10, 50 and 90% of the repertoire.

Supplementary Table 1

Network property	Definition (unit*)	Illustration
**Node (vertex)**	The fundamental unit of which graphs are formed: v	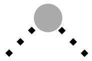
**Edge (link)**	An unordered pair of distinct vertices: $\{v, w\}$	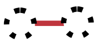
**Degree**	The number of edges incident to a vertex e: $deg(v)$	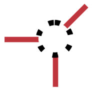
**Largest component**	Largest subgraph in which any two vertices are connected	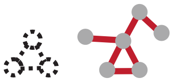
**k-core**	A maximal subgraph of a graph in which all vertices have degree of at least k	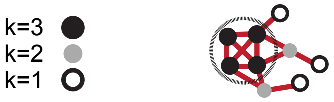
**Clique**	A complete subgraph in a graph	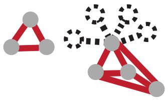
**Diameter**	The length of the "longest shortest path" between any two vertices: $\max_{(v,w)} d(v, w)$	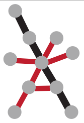
**Assortativity coefficient**	Pearson correlation coefficient of degree between pairs of linked nodes $r = \{-1,1\}$	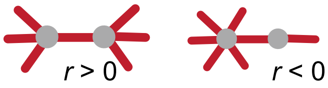
**Cluster** size, number	Connected component of a graph in which any two nodes are connected	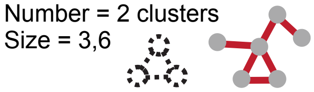
**Clustering coefficient (transitivity)**	The probability that the adjacent vertices of a vertex are connected	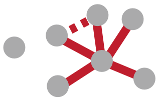
**Density**	The ratio of the number of edges and the number of possible edges	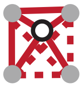
**Centralization**	Centrality score based on node-level centrality c: $\mathrm{sum}(\max(c(w), w) - c(v), v)$	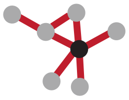
**Average Degree**	The average number of degrees per node: $2e/v$	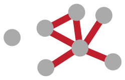
**Neighborhood**	Set of all the nodes that are adjacent to a node v: $N(v)$	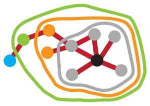

*These properties are numeric and dimensionless.

Supplementary Table 1. Network global properties.

Supplementary Table 2

Network property	Definition*	Illustration
**Eigenvector**	Principal eigenvector of *t(A)*A*, where *A* is the adjacency matrix of the graph: $x_v = \frac{1}{\lambda}\sum_{t\in M(v)} x_t$	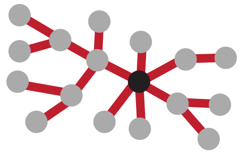
**Authority**	Principal eigenvector of *t(A)*A*, where A is the adjacency matrix of the graph	
**PageRank**	Principal eigenvector of the normalized matrix of the graph	
**Closeness**	Node centrality in a graph: $C(v) = \frac{1}{\sum_w d(v,w)}$	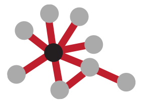
**Betweenness**	Number of shortest paths through *v*: $B(v) = \sum_{s\neq v\neq t} \frac{\delta_{st}(v)}{\delta_{st}}$	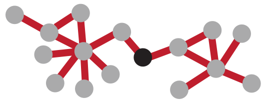

Supplementary Table 2. Network local properties. *These properties are dimensionless.

Supplementary Table 3

Human Sample	CDR3s (nodes/graph)
D1-M	2’305’669
D2-M	1’836’019
D3-M	3’127’059
D1-Na	6’187’146
D1-Nb	5’716’124
D2-N	4’408’661
D3-N	6’348’502

Supplementary Table 3. Human memory and naïve B cell network size.
Size of memory (n=3) and naïve B-cell repertoire (n=4) networks.
